# Wearable Emotion Recognition Using Heart Rate Data from a Smart Bracelet

**DOI:** 10.3390/s20030718

**Published:** 2020-01-28

**Authors:** Lin Shu, Yang Yu, Wenzhuo Chen, Haoqiang Hua, Qin Li, Jianxiu Jin, Xiangmin Xu

**Affiliations:** 1 School of Electronic and Information Engineering, South China University of Technology, Guangzhou 510641, China; shul@scut.edu.cn (L.S.); 201720212333@mail.scut.edu.cn (Y.Y.); 201821011745@mail.scut.edu.cn (W.C.); 201810101923@mail.scut.edu.cn (H.H.); xmxu@scut.edu.cn (X.X.); 2 Institute of Modern Industrial Technology of SCUT in Zhongshan, Zhongshan 528400, China; 3 School of Software Engineering, the Shenzhen Institute of Information Technology, Shenzhen 518172, China; liqin@sziit.edu.cn

**Keywords:** emotion recognition, smart bracelet, heart rate, wearable

## Abstract

Emotion recognition and monitoring based on commonly used wearable devices can play an important role in psychological health monitoring and human-computer interaction. However, the existing methods cannot rely on the common smart bracelets or watches for emotion monitoring in daily life. To address this issue, our study proposes a method for emotional recognition using heart rate data from a wearable smart bracelet. A ‘neutral + target’ pair emotion stimulation experimental paradigm was presented, and a dataset of heart rate from 25 subjects was established, where neutral plus target emotion (neutral, happy, and sad) stimulation video pairs from China’s standard Emotional Video Stimuli materials (CEVS) were applied to the recruited subjects. Normalized features from the data of target emotions normalized by the baseline data of neutral mood were adopted. Emotion recognition experiment results approved the effectiveness of ‘neutral + target’ video pair simulation experimental paradigm, the baseline setting using neutral mood data, and the normalized features, as well as the classifiers of Adaboost and GBDT on this dataset. This method will promote the development of wearable consumer electronic devices for monitoring human emotional moods.

## 1. Introduction

Emotions can significantly impact on our daily lives and work. Not only can emotions reflect a person’s mental state, but they also present a strong connection with people’s physical health [1]. Negative emotions have become key factors affecting human health. Studies have shown that long-term negative emotions can lead to various health problems such as headaches, asthma, ulcers, and heart diseases [2]. Due to the lack of diagnosis and treatment resources for psychological problems such as depression and anxiety in recent years, social problems are on the rise. Techniques for emotion recognition can improve human-computer interaction as well as psychological treatment to some extent [3]. Commonly used emotional recognition methods are based on behavioral parameters or physiological signals. Although emotional recognition based on behavior performance is intuitive and convenient, people can deliberately disguise emotional states in some situations. This can reduce reliability and accuracy. It is well known that physiological signals are affected by the human endocrine system and the autonomic nervous system. These systems are less affected by human subjective consciousness and can reflect the real emotional state more objectively and accurately [4]. From this perspective, emotion recognition based on physiological signals makes the results more objective.

Wearable emotion recognition devices using physiological signals have the potential for applications in our daily lives [5]. Some wearable devices are applied to people who are depressed or mentally handicapped to monitor their emotional states, as well as in the field of gaming. Currently, there are 300 million people with depression in the world, and predicting their mood can provide better care and prevent dangerous events. In the field of gaming, emotional changes can be used as an interactive means to change the game contents including scene and background music, so that players can have a better sense of immersion. It is hoped that more people will benefit from the wearable device based emotion recognition technology. Among the physiological signals, the heart rate is relatively easier for collection using various wearable devices such as a smart watch, bracelet, chest belt, and headset. At present, majorities of manufacturers have released smart bracelets or watch products with heart rate monitoring functions via photoplethysmography (PPG) sensors or electrocardiograph (ECG) electrodes. Devices from Apple, Huawei, Fitbit and Xiaomi provide a solid platform for wearable emotion recognition. We have tried to adopt a simple and effective way for daily emotional monitoring, so heart rate data captured by a smart bracelet is taken as the research object for comprehensive considerations. Moreover, heart rate is generated by the activity of the heart, controlled only by the human nervous system and endocrine system, and less affected by the subjective thinking. Human beings can hide emotions and do not show up in facial expressions and physical movements. However, heart rate characteristics due to emotions are difficult to control. Compared with facial expressions and limb movements, the heart rate based result is more objective and the actual emotions are not easy to be hidden.

Studies have shown that the heart rate varies with mood changes. In 1983, an experiment designed and conducted by Ekman et al. proved that physiological signals had unique responses to different emotions. The heart rate increased significantly when people were angry or scared but decreased significantly in a state of disgust [6]. Britton’s research showed that the heart rate during a happy mood was lower than that in a neutral mood [7]. Valderas showed that the effects of relaxation and fear on heart rate were significantly different, and the average heart rate during happiness was lower than that while in a sad state [8]. Using the IBPSO algorithm, Xu et al. collected ECG and heart rate signals for emotion recognition in which the highest recognition rate of sadness and joy was 92.10% [9]. Quiroz et al. used walking acceleration sensor data and heart rate data from a smart watch to predict the emotional state of the subject. Several time series and statistical methods were adopted to analyze changes in mood. It was found that the accuracy of the individual’s emotional recognition model was higher than the individual’s baseline level, and the classification accuracy for happiness and sadness was higher than 78% [10]. Pollreisz et al. used a smart watch to collect data on electrodermal activity (EDA), skin temperature (SKT), and heart rate (HR) for ten subjects. All of the subjects were asked to fill out the Self–Assessment Manikin (SAM) form after watching an emotional stimuli video. They built a simple solution for emotion recognition based on the peaks in the EDA signal. The success rates of their algorithm and the SVM + GA was 64.66% and 90%, respectively [11]. Zhang et al. conducted an experiment in which 123 subjects were asked to wear smart bracelets with built-in accelerometers. They attempted to identify emotions from walking data using the LibSVM algorithm. They achieved classification accuracy of 91.3% (neutral vs. angry), 88.5% (neutral vs. happy), and 88.5% (happy vs. sad). The recognition rate of the three emotions (neutral, happy, and angry) achieved an accuracy of 81.2%. These results demonstrated that emotion could be reflected to some extent in walking, and wearable smart devices could be used to recognize human emotions [12]. Covello, et al. collected subjects’ ECG data through wearable wireless sensors for detecting human emotions. The proposed CDR (a basic emotion response, known as cardiac defense response) algorithm detected changes in non-stationary transitions which might indicate abrupt changes in heart rate regulation (specifically autonomic nervous system regulation) due to a fear or startle event. It achieved an overall accuracy of 65% on 40 subjects [13].

Table 1 summarizes the studies of wearable devices for measuring emotion recognition. Many are complex and time-consuming for real applications. To make the emotion recognition feasible on wearable devices, our study proposed a method for recognizing emotional states (happy, sad, and neutral) of subjects via the heart rate signals from a wearable bracelet.

## 2. Experiment

### 2.1. Subject Information

A total of 25 subjects (Chinese, 13 females and 12 males) were recruited for the experiment. They ranged in age from 22–25 years old with an average of 23.5 years [16,17,18,19]. All the subjects were in good health, without any psychiatric illnesses, and free of any alcohol or medications that could delay the emotional response within the prior 72 h. A written consent was obtained from each subject prior to the experiment. All of the procedures were approved by the Research Ethics Committee of the Body Data Science Engineering Center in Guangdong Province, China (BDS18-06).

### 2.2. Stimulation Materials

**Figure 1:**
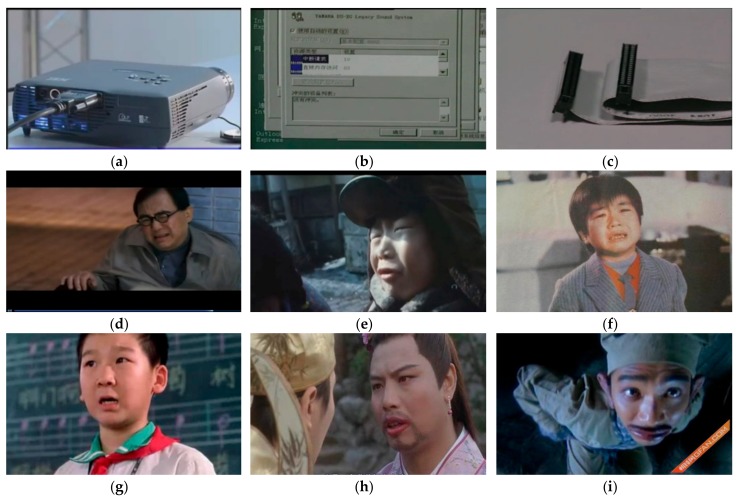
Scenes from the selected videos with its corresponding emotional response: (**a**–**c**) neutral; (**d**–**f**) sad; (**g**–**i**) happy. CEVS permission obtained [20].

This experiment relied on videos as the sources of emotional stimulus materials to induce corresponding emotions. Fifteen videos were selected from the CEVS (China’s Standard Emotional Video Stimuli Materials Library) and portrayed three categories of emotions: neutral, happy, and sad, as can be seen in Figure 1 [20]. The length of the videos ranged from 53 s to 3 min, as shown in Table 2. The materials in this database passed standardized evaluation. 48 video clips of 6 different emotions, including happiness, sadness, anger, fear, disgust and neutrality, were collected. According to the length and comprehensibility of the clips, 30 video clips of emotions were selected, and 50 subjects had evaluated the video clips. Statistical analysis showed that in terms of arousal, the main effect of emotion type was significant (F = 23.232, *p* < 0.001) [20].

### 2.3. Experiment Process

**Figure 2:**
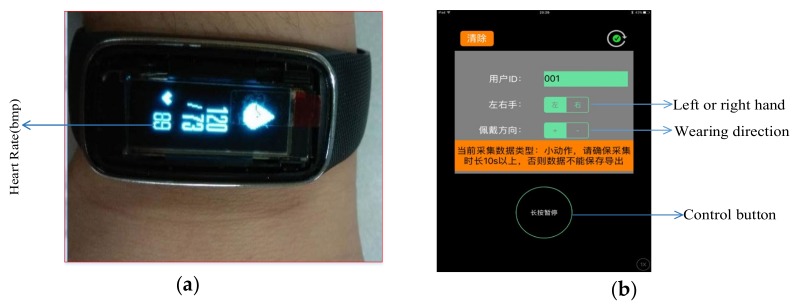
Experimental setup: The heart rate signal of the subject was collected using a smart bracelet (Algoband F8), and the sampling rate of the bracelet was 25 Hz: (**a**) The bracelet was worn on the subject’s wrist; (**b**) Connection and data recording APP via Bluetooth.

Before watching the videos, the subjects were informed about how the experiment would be conducted. The experiments were conducted in a 30-dB soundproof test room (Hengqi, Foshan, China). All of the subjects wore a smart bracelet (Algoband F8, Desay Electronics, Huizhou, China), and were required to watch three video pairs of ‘neutral + target emotion’ videos. The pair sets were design to evoke the following emotions: video set 1: neutral and neutral, video set 2: neutral and happy, and video set 3: neutral and sad. All the subjects were required to rest for 5 min at the beginning of the experiment to achieve a resting state. After each pair set of videos was shown, there was a minimum 5-min break to reduce the emotional interference of the previous video with the emotional response to the next video. In each set of videos, the first portion was neutral so that the subject could return to a neutral mood before viewing the mood stimulus material that followed, which was used to determine the baseline of the subject’s heart rate data. Physiological data were recorded along the time. The experimental setup is shown in Figure 2.

**Figure 3:**
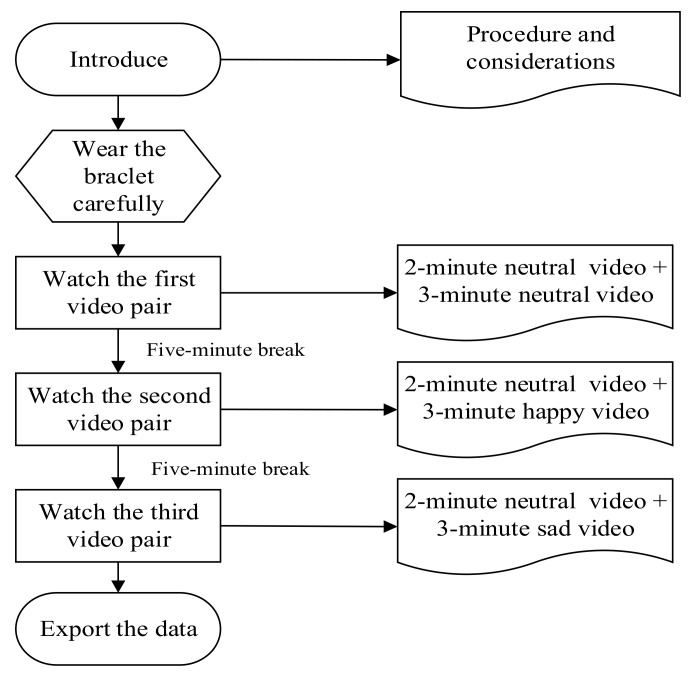
Steps of the experiment.

Figure 3 shows the flow chart of the experiment procedures. Before watching each video, a neutral emotion video was shown to let the subject return to a neutral state for reducing interference. The data extracted in the subject’s neutral state was used as the baseline heart rate.

The 2-video pair set used in our study reduced interference between different video induced emotions. There was a significant emotional difference between two portions in each video pair set (except the neutral-neural set), which made the subjects more likely to feel the change in emotional mood and therefore produced a more effective stimulation effect. Changes in heart rate during the videos were recorded and the data of the latter part of each video pairs were separated. Our main goal in the study was focusing on analyzing this part of the data. For each subject, the video pairs set (neutral + happy, neutral + sad, neutral+ neutral) were randomly selected in the material library CEVS, to avoid the data imbalance and emotional decay caused by using a single piece of material. All video materials in the experiment were professionally evaluated.

## 3. Data Processing

### Heart Rate Signal Pre-processing

**Figure 4:**
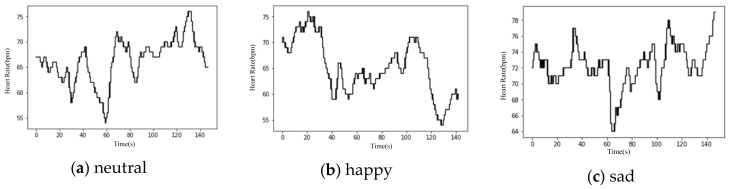
Typical heart rate data of the three stimulated emotional states (The horizontal axis is time in seconds and the vertical axis is heart rate in beats per minute): (**a**) neutral, (**b**) happy, (**c**) sad.

Each segment of the heart rate data was under two emotional states, where the first corresponded to the neutral state and the second was linked with the target emotion. Therefore, the corresponding original heart rate was selected in the latter part with reference to the length of each video in Table 1. Figure 4 shows the typical separated heart rate signals of one subject in the three stimulated emotional states.

Because of individual differences, the difference in baseline heart rates for each individual varied widely. To explore the subject-independent characteristics, we reduced the influence of individual differences [14,21]. Here, we defined the mean value of first part data under the neutral state as a baseline for removal of the individual difference. Therefore, the normalized heart rate can be calculated as the original data minus the baseline:

(1) $$Rate_{normal}=Rate_{original}-Rate_{neutral\_mean}$$

$Rate_{normal}$: the heart rate after reducing the influence of individual difference.

$Rate_{original}$: the original heart rate

$Rate_{neutral\_mean}$: baseline for removal of the difference in individual heart rates.

**Figure 5:**
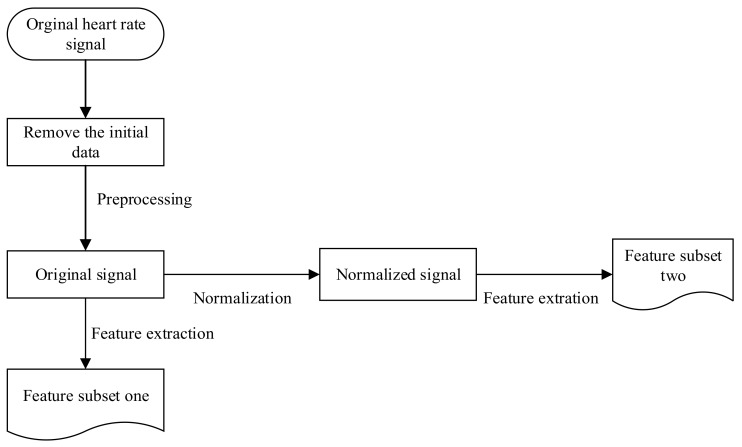
Steps of data processing.

Taking the average heart rate in the neutral state as the index value and subtracting that value from the original heart rate of the target emotion led to the normalized heart rate, as shown in Equation (1). The datasets that contribute to mood changes include commonality and personality. The former was extracted from the normalized heart rate. The latter was extracted from the original data to determine the variation in heart rate of the different moods. As shown in Figure 5, from the original heart rate data, the universal characteristics that reflect the emotional changes were first extracted from original data as the feature subset one, and the characteristics from the normalized signals that removed the individual differences were then extracted as the feature subset two [21].

## 4. Feature Extraction

**Figure 6:**
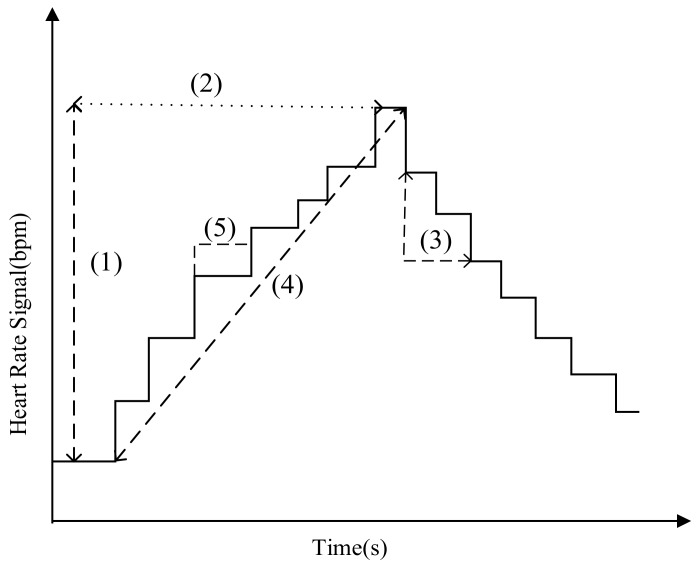
Typical Heart rate parameters for feature extraction: (**1**) Up_Amplitude. (**2**) Up_Time. (**3**) Down_Amplitude. (**4**) Slope. (**5**) T_Continue.

As changes in emotional mood cause changes in heart activity, the extracted features can be used to express the state of different emotions. Figure 6 shows some typical parameters used for feature extractions.

Parameters (1) and (3) represent the amplitude change of the heart rate. Parameter (2) indicates that the heart rate continues to rise in time and (4) shows the change slope of the heart rate. Parameter (5) denotes the duration when heart rate data remains unchanged. The features used in our study are divided into two parts: one from the original signal and the other from the normalized signal.

### 4.1. Features of the Original Signal

Rate_diff1_mean (Diff1) denotes the mean value of the first-order difference in heart rates. $X_{n}$ represents the heart rate from the original signal. N represents the total length of the discrete data [14,22]:

(2) $$D{\mathrm{iff}}_{1}=\frac{1}{N-1}\sum_{n=1}^{N-1}\left|X_{n+1}-X_{n}\right|$$

Rate_diff2_mean (Diff2) denotes the second-order difference in heart rates:

(3) $$D{\mathrm{iff}}_{2}=\frac{1}{N-2}\sum_{n=1}^{N-2}\left|X_{n+2}-X_{n}\right|$$

Rate_range (*H_range_*) denotes the variation range of the heart rate:

(4) $$H_{range}=Heart_{\max}-Heart_{\min}$$

Rate_data_entropy denotes the information entropy of the heart rates. It indicates the degree of dispersion of heart rate data,$x_{i}$ represents the value of the heart rate [23].

(5) $$H(X)=-\sum_{i=1}^{n}p(x_{i})\log p(x_{i})$$

Max_ratio (Ratio_max) denotes the ratio of the maximum heart rate value and data length:

(6) $$Ratio\_\max=\frac{Heart\_rate_{\max}}{N}$$

Min_ratio (Ratio_min) denotes the ratio of the minimum heart rate value and data length:

(7) $$Ratio\_m\mathrm{in}=\frac{Heart\_rate_{\min}}{N}$$

Rate_Adjacent_data_root_mean (Radrm) denotes the root means square of the difference between adjacent heart rate data elements in a sequence [4]:

(8) $$Radrm=\frac{\sum_{i=1}^{N}{\left(X_{i+1}-X_{i}\right)}^{2}}{N}$$

Rate_Down_Time describes the time when the heart rate decreases, and its specific characteristics include the following:

*Rate_down_time_max, Rate_down_time_min, Rate_down_time_median, Rate_down_time_mean, Rate_down_time_std*.

Rate_Up_Time describes the time that heart rate increases. The max, min, median, mean, and variance of this feature were calculated to describe its characteristics, which were noted as: *Rate_up_time_max*, *Rate_up_time_min*, *Rate_up_time_median*, *Rate_up_time_mean*, *Rate_up_time_std*.

Rate_Time_continue denotes the duration when heart rate data remains unchanged. The five statistical characteristics are

*Rate_time_continue_max, Rate_time_continue_min, Rate_time_continue_median, Rate_time_continue_std, and Rate_time_continue_mean*.

### 4.2. Features of the Normalized Signal

Rate_Down_Slope can be calculated using Equation (9), where *Down_amplitude_* represents the amplitude decline of the normalized heart rate, *Down_time_* represents the corresponding decrease time. Its specific characteristics include:

*Rate_down_slope__max, Rate_down_slope__min, Rate_down_slope_ mean, Rate_down_slope_ median, and Rate_down_slope_std*.

(9) $$Down\_s\mathrm{lo}pe=\frac{Down_{amplitude}}{Down_{Time}}$$

Rate_amplitude_var denotes the variance of normalized heart rate data.

Rate_up_amplitude represents the amplitude change when the normalized heart rate increases, and its specific characteristics include

*Rate_up_amplitude_max, Rate_up_amplitude_median, Rate_up_amplitude_mean, and Rate_up_amplitude_std*.

Rate_down_amplitude represents the amplitude change when the normalized heart rate declines, and the five statistical characteristics are

*Rate_down_amplitude_max, Rate_down_amplitude_min, Rate_down_amplitude_median, Rate_down_amplitude_mean, and Rate_down_amplitude_std*.

**Figure 7:**
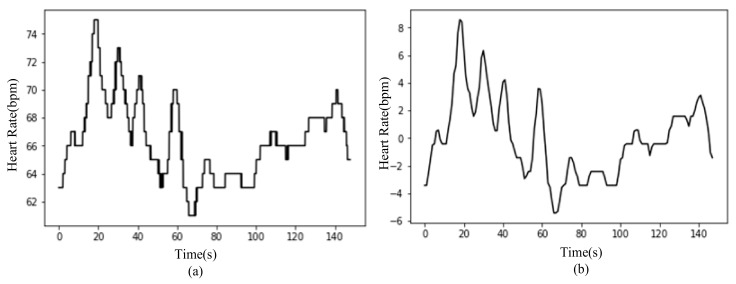
Normalized signal and its 25-mean data: (**a**) normalized signal, (**b**) 25_mean data.

The normalized signal is processed by moving average with a window length of 25, then the 25_mean data is obtained, as seen in Figure 7. It mainly includes the following five characteristics of *25_mean_max*, *25_mean_min*, *25_mean_median*, *25_mean_mean*, *25_mean_std* [11,12].

*Rate_data_mean*, *Rate_data_var* represents the mean and variance of the normalized heart rate signal $\overset{\text{\textasciitilde{}}}{X}$ respectively.

(10) $$Rate\_data\_mean=\frac{1}{N}\sum_{i=1}^{N}\overset{\text{\textasciitilde{}}}{X_{i}}$$

(11) $$Rate\_data\_\mathrm{var}=\frac{1}{N-1}\sum_{i=1}^{N}\left(\overset{\text{\textasciitilde{}}}{X_{i}}-mean(\overset{\text{\textasciitilde{}}}{X})\right)$$

*Rate_diff1_normalization* (Diff1_normalization) denotes the average of the first-order difference of the normalized original signals [12,14]. It contains the following five characteristics:

*Rate_data_normazation_diff1_max, Rate_data_normazation_diff1_min, Rate_data_normazation_diff1_std, Rate_data_normazation_diff1_median, Rate_data_normazation_diff1_mean*:

(12) $$D{\mathrm{iff}}_{1}\_normalized=\frac{1}{N-1}\sum_{n=1}^{N-1}\left|{\overset{\text{\textasciitilde{}}}{X}}_{n+1}-{\overset{\text{\textasciitilde{}}}{X}}_{n}\right|$$

*Rate_diff2_normalization* (Diff_2__normalization) denotes the average absolute value of the second-order differences of the normalized heart rate signals. Similarly, the following five features are also extracted:

*Rate_data_normazation_diff2_max, Rate_data_normazation_diff2_min, Rate_data_normazation_diff2_std, Rate_data_normazation_diff2_median, Rate_data_normazation_diff2_mean*:

(13) $$D{\mathrm{iff}}_{2}\_normalized=\frac{1}{N-2}\sum_{n=1}^{N-2}\left|\overset{\text{\textasciitilde{}}}{X_{n+2}}-\overset{\text{\textasciitilde{}}}{X_{n}}\right|$$

## 5. Selection of Features

As shown in Table 3, 53 features were extracted from each piece of data. Explanation of features terms was supplemented in Table A1. To make the emotion recognition process easier, we needed to reduce the dimension of features by selection of the most effective features from total 53 features. We chose to adopt the SelectKBest for feature selection, which returned the top k features under an evaluation parameter setting of mutual_info_classif (classification problem). The SelectKBest used here was a library function of sklearn, which was a machine learning library implemented in Python. In this paper its corresponding version was 0.19.1. Anaconda (an open source Python distribution) was adopted, and sklearn was upgraded to the corresponding version through pip or CONDA instructions.

Table 4 shows the top five rated features with their corresponding scores for identifications of features that are important to each emotional category. The features of data_mean, 25_mean_median, data_entropy play the most important role in two or three emotional classifications, which are relevant to the mean and median amplitude value of the normalized signals and the information entropy of the original signals. Generally, it can be seen the features extracted from the normalized signals obtained relatively higher scores, such as data_mean, 25_mean, and data_normalized_diff1 series features, indicating the normalized heart rate data has a greater impact on the classification. The fusion of features from normalized and original signals might effectively improve the accuracy of recognition.

## 6. Model Training

To distinguish among the three emotional states (happy, sad, and neutral), five classifiers were evaluated including KNN (k-Nearest Neighbor) [15,24], RF (Random Forests) [5,25], DT (Decision Tree) [12,26], GBDT (Gradient Boosting Decision Tree) [27], and AdaBoost (Adaptive Boosting) [28]. We adopted these classifier models from the sklearn library, which integrated common machine learning methods. In each evaluation, there were 50 samples with two different emotional tags (25 subject data/emotion), and 75 samples in three emotional classifications [16]. “leave-one-out cross-validation” was used. Loop each data as a test set and the rest as a training set [29]. At the end of each round of operation, the correct samples were statistically counted to calculate the accuracy. The prediction values of all samples were evaluated and the predictive effect of the model was then calculated. Table 5 shows the parameter information of the classifiers. Classifier parameter definition was supplemented in Table A2. The accuracy rate calculated by Equation (14) was used to evaluate the effect of classification on different models, where *N_correct_* represents the number of samples that are correctly identified, and *N_total_* represents the total number of samples [16,22,27,30,31,32]:

(14) $$Accuracy=\frac{N_{correct}}{N_{total}}$$

## 7. Results

Our study calculated the accuracy of two and three emotional classifications to evaluate the performance of the classifiers. As stated in Section 5, we used the SelectKBest for feature selection and found that in many cases only the scores of the top 21 features were beyond zero. Therefore, k was set below 21 as 20, 16, 12, 10, 8, or 5. And the models’ performance was evaluated with various k settings. We used “leave-one-out cross-validation” here, and ten runs were conducted. The mode of the ten runs results was used as the final accuracy. Selecting the mode as the accuracy rate was conducive to predicting the approximate rate in a single experiment in the future. Actually, it was found that the average accuracy rate was very close to the mode value in the experiment. Next, the analysis of the classifier was mainly based on the comparison of mode results, which reflected the performance comparison of the classifier in recognition under the condition of high probability. The comparison results based on the “leave-one-out cross-validation” and the mode value could reflect the performance difference of the classifiers to some extent.

### 7.1. Categories of Neutral and Happy Emotions

**Figure 8:**
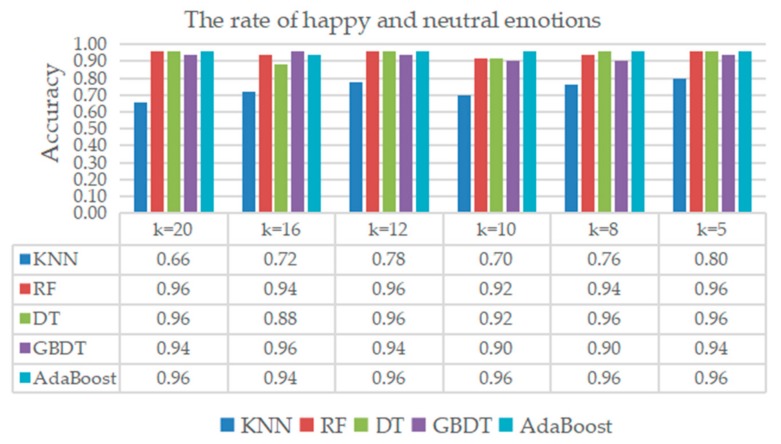
Classification result of happy and neutral emotions.

The recognition result of neutral and happy emotions is given in Figure 8. Except KNN, the accuracies of the RF, DT, GBDT, and the AdaBoost were all over 0.80, where AdaBoost ranked the first, whose average accuracy was around 0.96 under different k configurations.

### 7.2. Categories of Neutral and Sad Emotions

**Figure 9:**
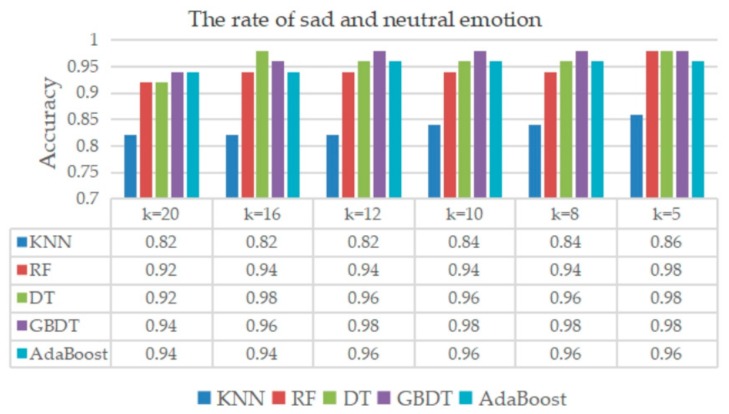
Classification result of sad and neutral emotions.

The classification result of neutral and sad emotions is shown in Figure 9, where the accuracy was over 0.8 on the five classifiers, and the classifiers of RF, DT, GBDT, and AdaBoost exhibited adequate performances.

### 7.3. Categories of Happy and Sad Emotions

**Figure 10:**
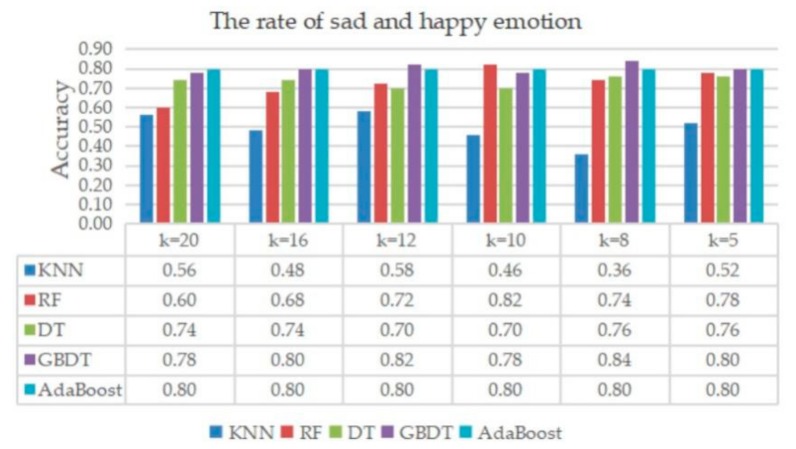
Classification result of happy and sad emotions.

Figure 10 shows the classification results of happy and sad emotions, where the accuracy rate was obviously lower than the previous two cases. The best performing classifier model was GBDT, which achieved a score of 0.84 at k = 8. The AdaBoost also achieved an acceptable rate of 0.80.

### 7.4. Classification of Three Emotions

**Figure 11:**
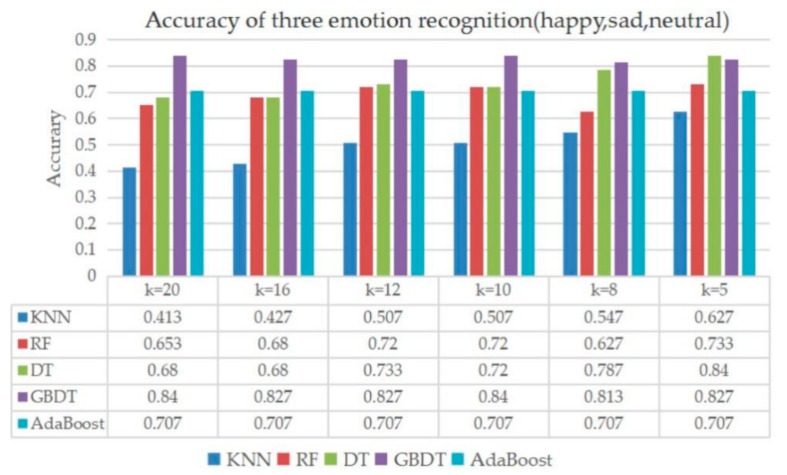
Classification result of neutral, happy and sad emotions.

In addition to the research of the two emotional classifications, this paper analyzed the classification performance of three different emotions (happy, neutral, and sad). Except KNN which adopted the parameter settings in Table 5, the other classifiers used the default configurations. The results in Figure 11 showed the best performing classification model was GBDT, which achieved a rate of 0.84 (k = 10). Considering all the two and three emotional classifications, the GBDT and AdaBoost algorithm were recommended which could achieve a better accuracy rate in our dataset.

## 8. Discussion

Our study proved that when human emotions were experienced and changed (happy, sad, and neutral) the heart rate could reflect the mood accordingly [5]. It also showed that real-time emotional recognition and monitoring could be achieved with the available wearable devices. This method was simple, quick, and easy to deploy on many available wearable devices. Several key issues are discussed below.

Experimental paradigm: we presented a way of ’neutral + target’ pair emotion stimulation experimental paradigm. Except the regular rest period between two emotional stimulation which let the subjects enter the resting state, the neutral stimulation video was added as the first part of each video pair as a control, which was conducive to the induction of target emotion. We then used the data of neutral mood as a baseline, and the results approved this baseline setting was applicable. For real emotion recognition applications by smart bracelet, we suggest standard neutral stimulation videos be firstly presented to the wearer, to obtain the heart rate data of neutral mood as a baseline. This operation helps to eliminate personalized differences.

Baseline operation: the experimental results showed that the features extracted from normalized signals contributed more to emotion recognition, which further validated the effectiveness of the baseline operations. Some literature adopted resting mood data as a baseline. It is worth further studying the merits and demerits of eliminating individual differences by using resting state or neutral state as baseline.

As for the classifiers, the recognition accuracies of the RF, DT, GBDT, and AdaBoost were higher than that of the KNN. Maybe it’s because the relationship between emotions and body physiological signatures (one to one, one to many, or many to many) has not been confirmed yet. Therefore using the classifiers based on the tree model are more suitable here.

Performance comparison: compared with the research on emotion recognition based on heart rate, we also achieved adequate recognition accuracy. Guo’s study [19] used a wearable electrocardiograph to collect single-lead ECG signals with a sampling frequency of 200 Hz. They conducted feature processing using PCA (principal component analysis) method, and selected five significant characteristic values to classify emotional states. 13 HRV features were used to classify two and five emotional states, and the accuracy was 70.4% and 52% respectively. If the accuracy was used as the evaluation, our method demonstrated better performance. Moreover, our work directly collected data through the wearable bracelet, which was simple and had certain application significance.

Sample size: the subject number of 25 was chosen with reference to the literature. The subject number of the following referenced studies was also around 25, which indicated 25 subjects can reflect some feature to some extent. The DEAP was a standard database for emotion recognition research based on multi-channel physiological signals and facial expressions, which collected physiological signals from a total of 32 subjects. Many researchers had conducted experiments on emotion recognition based on the database [17]. In Guo’s study [22], HRV was used for emotion recognition, and physiological data of 25 healthy people aged 29 to 39 were collected, which achieved good recognition result. In another reference [19], researchers collected data from 21 healthy subjects using a wearable ECG device, where short-term HRV data were analyzed for mood recognition. The SEED dataset was another standard collection of EEG data for emotion recognition from 15 subjects [18]. Although our sample size of 25 was similar to the previous studies, it might not be representative of population characteristics, and this method was announced to be effective on this dataset. The applicability of this method to other data sets deserves further exploration.

Activity effect: daily life activities will affect the accuracy in heart rate monitoring based on the bracelet, due to the slip, friction and sweat between human skin and the bracelet. In particular, motion artifact will increase the measurement noises and even the situations that the accurate data cannot be obtained. To avoid these influences, we suggest the bracelet be tightly worn on the wrist, and the mechanism of activity monitoring by the bracelet integrated motion sensors be adopted. Therefore, only the heart rate data under static (such as sleeping, standing, and sitting) or quasi-static (such as jogging) states can be used in daily emotion monitoring, while the data of dynamic states (such as running) are not used. This mechanism can lead to a rough assessment of emotions throughout the day. A more accurate analysis depends on the improvement of the wearable dynamic heart rate monitoring. It deserves further studies on minimizing the activity effect in heart rate-based daily mood monitoring.

PPG effect: we used the smart bracelet with PPG to collect the pulse rate at the wrist in this study. While in the absence of major diseases, the pulse rate is equal to the heart rate, the heart rate collected by PPG is susceptible to the influence of motion artifacts. Therefore, during the experiment, subjects were required to stay still and wore the smart bracelet as tight as possible to reduce the interference of motion artifacts. Noise reduction algorithms were embedded to reduce the interference caused by movement to a certain extent. Additionally, as the interference caused by blood pressure to PPG sensor also contained certain emotional information [33], it was also included for final emotion estimation.

Furthermore, this study used standard emotional stimulation videos to bring passive induced emotion to the subjects. However, in real life, human emotions often include active and passive induced emotions. Whether there is difference between the physiological representations of active emotion and passive emotion is still worth further study.

The duration of stimulation, the rest period and the interval among different emotion stimulation were all determined according to the literature and experience. However the cumulative and attenuating effects of emotions have not been completely confirmed yet. And due to individual differences, the group norm of these parameters has not been established yet, which deserves further studies.

## 9. Conclusions

In our study, we proposed a method of using heart rate data to identify human emotions. The data was collected by a wearable smart bracelet. The experimental results showed that our method was an effective means of using the heart rate signal to recognize human emotions. This method is simple to be realized on wearable consumer electronic devices. It will help to promote the application and development of wearable devices for monitoring human emotional moods in static or quasi-static states.

## Figures and Tables

**Table 1:** Summary of related research.

Ref.	Related Work	Signal Type	Subject Number	Stimulation Materials	Accuracy
[10]	Quiroz, J.C., Geangu, E., & Yong, M.H. (2018)	Walking sensor data and heart rate data	50	Audio-visual and audio	Higher than 78% (happiness vs. sadness)
[11]	David Pollreisz and Nima Taheri Nejad (2008)	EDA, SKT, HR	10	Emotion video	64.66% (simpler)
[12]	Zhan Zhang et al. (2016)	Walking data	123	Film chip	91.3% (neutral vs. angry), 88.5% (neutral vs. happy), 88.5% (happy vs. angry)
[14]	Xu Ya (2010)	ECG, HR	300	Video	Happy (80.38%)
[15]	Tengfei Song et al. (2019)	ECG	23	Video	Joy, sad, neutral (50.66%)

**Table 2:** Duration of the Selected Videos (seconds).

Emotion	Video 1	Video 2	Video 3	Video 4	Video 5
**Neutral**	74	70	68	122	71
**Happy**	53	109	142	83	112
**Sad**	233	146	88	136	101

**Table 3:** List of features.

Categories	Features
Features from original signal	1. *Rate_diff1_mean*, *Rate_diff2_mean*, *Rate_range*, *Rate_data_entropy*, *Max_ratio*, *Min_ratio*, *Rate_Adjacent_data_root_mean.* 2. *Rate_down_time_max*, *Rate_down_time_min*, *Rate_down_time_median*, *Rate_down_time_mean*, *Rate_down_time_std.* 3. *Rate_up_time_max*, *Rate_up_time_min*, *Rate_up_time_median*, *Rate_up_time_mean*, *Rate_up_time_std.* 4. *Rate_time_continue_max*, *Rate_time_continue_min*, *Rate_time_continue_median*, *Rate_time_continue_mean*, *Rate_time_continue_std.*
Features from normalized signal	1. *Rate_down_slope_max*, *Rate_down_slope_min*, *Rate_down_slope_median*, *Rate_down_slope_mean*, *Rate_down_slope_std.* 2. *Rate_up_amplitude_max*, *Rate_up_amplitude_median*, *Rate_up_amplitude_mean*, *Rate_up_amplitude_std.* 3. *Rate_down_amplitude_max*, *Rate_down_amplitude_min*, *Rate_down_amplitude_median*, *Rate_down_amplitude_mean*, *Rate_down_amplitude_std.* 4. *25_mean_max*, *min*, *median*, *mean*, *std.* 5. *Rate_data_mean*, *Rate_data_var.* 6. *Rate_data_normalized_diff1_max\min\std\median\mean.* 7. *Rate_data_normalized_diff2_max\min\std\median\mean.*

**Table 4:** Scores of the Top Five Features.

Emotional Categories	Feature Name	Feature Score
Neutral vs. happy	data_mean	0.5598
	25_mean_median	0.5063
	25_mean_mean	0.3002
	data_entropy	0.2655
	data_normalized_diff1_median	0.1376
Neutral vs. sad	25_mean_median	0.5119
	data_mean	0.5119
	25_mean_mean	0.3368
	25_mean_max	0.1392
	slope_max	0.1158
Happy vs. sad	data_entropy	0.3941
	down_amplitude_max	0.1345
	down_time_mean	0.1149
	data_normalized_diff1_max	0.0896
	down_amplitude_min	0.0879
Sad, Happy, Neutral	data_mean	0.4912
	25_mean_median	0.4612
	data_entropy	0.3206
	25_mean_mean	0.2967
	slope_max	0.1106

**Table 5:** Classifier Parameters.

Classifier	Parameter
KNN	weights = ‘distance’, p = 1, n_neighbors = 6, leaf_size = 2, algorithm = ‘ball_tree’
RF	n_estimators = 90, oob_score = True, random_state = 10
DT	criterion = ‘gini’, max_depth = 6, splitter = ‘best’
GBDT	n_estimators = 120, max_depth = 10, learning_rate = 0.01,min_samples_split = 4, subsample = 0.5
AdaBoost	n_estimators = 6, learning_rate = 0.1

## Appendix A

**Table A1:** Explanation of features terms.

Categories	Features Explanation
Features from original signal	*Rate_diff1_mean*: the mean value of the first-order difference in heart rates; *Rate_diff2_mean*: the mean value of the second-order difference in heart rates; *Rate_range*: the variation range of the heart rate; *Rate_data_entropy*: the information entropy of the heart rates; *Max_ratio*: the ratio of the maximum heart rate value and data length; *Min_ratio*: the ratio of the minimum heart rate value and data length; *Rate_Adiacent_data_root_mean*: the root means square of the difference between adjacent heart rate data elements in a sequence; *Rate_down_time_max*: the max value during the time when the heart rate decreases; *Rate_down_time_min*: the min value during the time when the heart rate decreases; *Rate_down_time_median*: the median value during the time when the heart rate decreases; *Rate_down_time_mean*: the mean value during the time when the heart rate decreases; *Rate_down_time_std*: the standard deviation during the time when the heart rate decreases; *Rate_up_time_max*: the max value during the time when the heart rate increases; *Rate_up_time_min*: the min value during the time when the heart rate increases; *Rate_up_time_median*: the median value during the time when the heart rate increases; *Rate_up_time_mean*: the mean value during the time when the heart rate increases; *Rate_up_time_std*: the standard deviation during the time when the heart rate increases; *Rate_time_continue_max*: the max value during the time when the heart rate remains unchanged; *Rate_time_continue_min*: the min value during the time when the heart rate remains unchanged; *Rate_time_continue_median*: the median value during the time when the heart rate remains unchanged; *Rate_time_continue_mean*: the mean value during the time when the heart rate remains unchanged; *Rate_time_continue_std*: the standard deviation during the time when the heart rate remains unchanged;
Features from normalized signal	*Rate_down_slope_max*: the max value of the ratio of decreased amplitude and the corresponding decrease time; *Rate_down_slope_min*: the min value of the ratio of decreased amplitude and the corresponding decrease time; *Rate_down_slope_median*: the median value of the ratio of decreased amplitude and the corresponding decrease time; *Rate_down_slope_mean*: the mean value of the ratio of decreased amplitude and the corresponding decrease time; *Rate_down_slope_std*: the standard deviation of the ratio of decreased amplitude and the corresponding decrease time; *Rate_up_amplitude_max*: the max value of the amplitude change when the normalized heart rate increases; *Rate_up_amplitude_median*: the median value of the amplitude change when the normalized heart rate increases;*Rate_up_amplitude_mean*: the mean value of the amplitude change when the normalized heart rate increases; *Rate_up_amplitude_std*: the standard deviation of the amplitude change when the normalized heart rate increases; *Rate_down_amplitude_max*: the max value of the amplitude change when the normalized heart rate declines; *Rate_down_amplitude_min*: the min value of the amplitude change when the normalized heart rate declines; *Rate_down_amplitude_median*: the median value of the amplitude change when the normalized heart rate declines; *Rate_down_amplitude_mean*: the mean value of the amplitude change when the normalized heart rate declines; *Rate_down_amplitude_std*: the standard deviation of the amplitude change when the normalized heart rate declines; *25_mean_max, min, median, mean, std*: the normalized signal is processed by moving average with a window length of 25, then the 25_mean data is obtained. Then features (includes max, min, median, mean, std) are extracted from the 25_mean data;*Rate_data_mean,Rate_data_var*: the mean and variance of the normalized heart rate signal; *Rate_data_normalized_diff1_max\min\std\median\mean*: Rate_data_normalized_diff1 is the average of the first-order difference of the normalized original signals. Then features (includes max, min, std, median, mean) are extracted from Rate_data_normalized_diff1 data; *Rate_data_normalized_diff2_max\min\std\median\mean*: Rate_data_normalized_diff2 is the average absolute value of the second-order differences of the normalized heart rate signals. Then features (includes max, min, std, median, mean) are extracted from Rate_data_normalized_diff2 data;

**Table A2:** Classifier parameter definition.

Classifier	Parameter	Parameter Explanation
KNN	weights = ‘distance’, p = 1, n_neighbors = 6, leaf_size = 2, algorithm = ‘ball_tree’	Weights: weight function used in prediction; p: power parameter for the Minkowski metric; n_neighbors: number of neighbors to use; leaf_size: leaf size passed to BallTree; algorithm: used to compute the nearest neighbors
RF	n_estimators = 90, oob_score = True, random_state = 10	n_estimators: the number of trees in the forest; oob_score: whether to use out-of-bag samples; random_state: controls both the randomness of the bootstrapping of the samples used when building trees and the sampling of the features to consider when looking for the best split at each node
DT	criterion = ‘gini’, max_depth = 6, splitter = ‘best’	criterion: the function to measure the quality of a split; max_depth: the maximum depth of the tree; splitter: the strategy used to choose the split at each node
GBDT	n_estimators = 120, max_depth = 10, learning_rate = 0.01, min_samples_split = 4, subsample = 0.5	n_estimators: the number of boosting stages to perform; max_depth: maximum depth of the regression estimators; learning_rate:learning rate shrinks the contribution of each tree by learning_rate; min_samples_split: the minimum number of samples required to split an internal node; subsample: the fraction of samples to be used for fitting the individual base learners
AdaBoost	n_estimators = 6, learning_rate = 0.1	n_estimators: the maximum number of estimators at which boosting is terminated; learning_rate: learning rate shrinks the contribution of each classifier
